# Simulation of settlement and bearing capacity of shallow foundations with soft particle code (SPARC) and FE

**DOI:** 10.1007/s13137-018-0109-z

**Published:** 2018-08-24

**Authors:** Barbara Schneider-Muntau, Iman Bathaeian

**Affiliations:** 1 Division of Geotechnical and Tunnel Engineering, University of Innsbruck, Technikerstr. 13, 6020 Innsbruck, Austria; 2 Present Address: ZSZ - Ingenieure ZT GmbH, Adolf-Pichler-Platz 12, 6020 Innsbruck, Austria

**Keywords:** Meshfree methods (SPARC), Finite element method, Shallow foundation, Bearing capacity, Hypoplasticity, Barodesy for clay, Shear band, 35D35, 35Q74, 65D05, 65M99, 74D10, 74C15

## Abstract

In this study we investigate the development of shear zones due to the settlement of shallow foundations and their load-settlement behavior. Firstly, a well-documented experiment of shallow penetration into sand is used for the validation of the soft particle code (SPARC). For these simulations a hypoplastic material model for sand with calibration for the model sand is implemented in SPARC. In order to deliver a more comprehensive investigation, the shape of the shear zones predicted by SPARC is also compared with the analytical solution. Secondly, the penetration of shallow foundation into clay is investigated by means of SPARC and the finite element method. For this purpose, barodesy for clay with the calibration for Dresden clay is implemented in both numerical methods. The simulations are carried out for six different surcharges, corresponding to a range of over-consolidated clay to normal-consolidated clay. Furthermore, the load-settlement behavior and the shape of shear zones for both methods are compared and the weaknesses and strengths of each numerical approach are discussed. Finally, the peaks of the load-settlement curves for all surcharges are compared with the analytical solution. Results show that SPARC performs better at predicting the trajectories of particles under the foundation, which consequently leads to better estimation of the load-settlement behavior.

## Introduction

Since the emergence of the FE methods, numerical methods have become the so-called *third pillar* besides theory and experiment in understanding the response of structures and analysis of engineering problems. Like any other field of mechanical engineering, geotechnical engineering and soil mechanics also benefit from the numerical methods in predicting the soil deformations under different loading patterns. One of the shortages of most numerical methods is the inability to simulate large deformations due to settlements, installation processes (e.g. penetration) and excavation, therefore, the simplified *wished-in-place* method is vastly employed in numerical simulations. One main drawback of wished-in-place method is that the deformations of the installation process are neglected, which leads to an underestimation of the deformations and consequently the serviceability and in more serious cases even the stability of the buildings and infrastructures are endangered. Several studies have proven that the final deformations in geotechnical projects have been larger than those estimated by the numerical methods and the unforeseen deformations are mainly caused during the installation phases, e.g. Triantafyllidis (1998). However, settlements due to the penetration of shallow foundations cannot be easily simulated by means of numerical methods, since they are associated with large and non-topological deformations. The FE methods need to retreat to remeshing strategies in order to avoid numerical problems. In contrast to FE methods, meshfree methods have no fixed connectivities between the points and are therefore more appropriate for problems associated with penetration. In this study we use the conventional FE method (Abaqus) with a user defined material model [Barodesy for Clay Medicus et al. (2016) and Medicus and Fellin (2017)] implemented as UMAT (Schneider-Muntau et al. 2017, 2018. The meshfree code (SPARC) applied in this study has been earlier introduced in Ostermann et al. (2013), Schneider-Muntau et al. (2017), Michel et al. (2017), Chen (2014) and Polymerou (2017).

This study focuses mainly on settlements and bearing capacity due to penetration of shallow foundations into sand and clay and investigates the formation of shear bands and load-settlement behavior. In Sect. 2 the failure mechanisms for shallow penetration are discussed and the ultimate bearing capacity equation is explained in its general form. In Sect. 3 an experimental shallow penetration test is introduced, which is applied in this study for the validation of the Soft PArticle Code (SPARC). In Sect. 4, the experiment is simulated with SPARC and the results are compared with PIV^1^ evaluations of the experiment. In Sect. 5, the same geometry is applied for investigation of formation of shear bands and load-settlement behavior of shallow foundations on clay. In Sect. 6, the results of simulations for SPARC and FE are compared and also the ultimate bearing capacities calculated by each method are compared with the analytical solution. Finally, in Sect. 7, the conclusion and final remarks are offered.

## Failure mechanisms

Due to the complicated nature of soils and the failure mechanisms associated with shallow penetration, derivation of mathematical solution for analysis of failure and the bearing capacity of shallow foundations is a not a simple task. All methods make simplifying assumptions regarding the *soil properties* (e.g. ideal rigid plastic material) and the *pattern of deformations* (e.g. formation of a wedge of soil under the foundation). Despite such simplifications, comparisons between model tests and full-sized foundations demonstrate that the acquired failure mechanisms for sand are comparable with real failure mechanisms (Heinz 1970; Muhs and Weiß 1972). However, in case of clayey soils, the failure mechanisms have not been thoroughly investigated and the pattern of shear zones are almost unknown (Tani and Craig 1995).

**Fig. 1:**
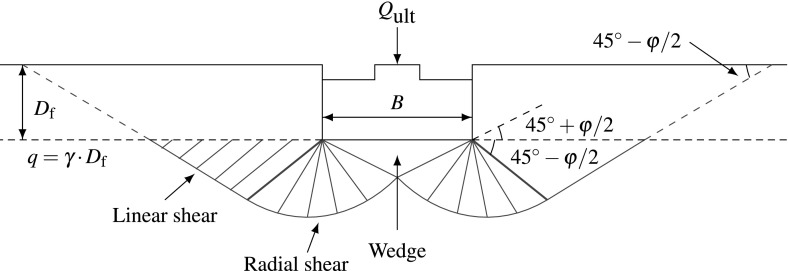
Shear failure of dense soil

Based on Mohr-Coulomb criterion, slip planes develop when soil is sheared to failure. However, rigid foundations avoid formation of any slip plane through the foundation and as a result, no slip plane is formed just below the foundation but only a rigid wedge. The wedge just below the foundation in Figs. 1 and 3 penetrates into the soil and causes twin zones of shear, in the first zone, adjacent to the wedge, radial shear planes are formed. In the neighboring triangular zone, linear shear planes are formed (Budhu 2000; Leonards 1962).

**Fig. 2:**
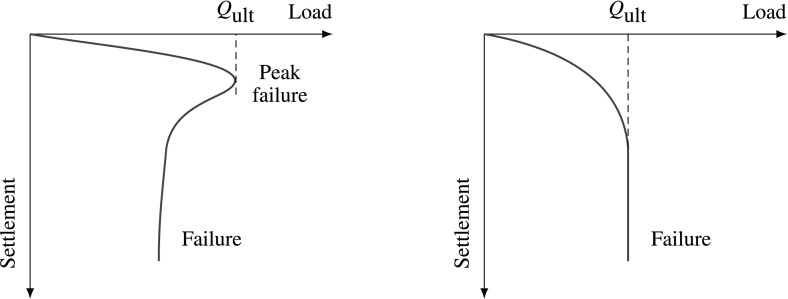
Load-settlement behavior—left: dense soil, right: loose soil

We need to distinguish here between failure mechanisms in dense soil and in loose soil, as for the dense dilating soil, the collapse corresponds to the peak friction angle $\varphi _{p}$, see the left diagram in Fig. 2. On the other hand, for loose non-dilating soil, the failure is associated with the critical friction angle $\varphi _{c}$ and no peak in load-settlement curve is expected, see the right diagram in Fig. 2.

**Fig. 3:**
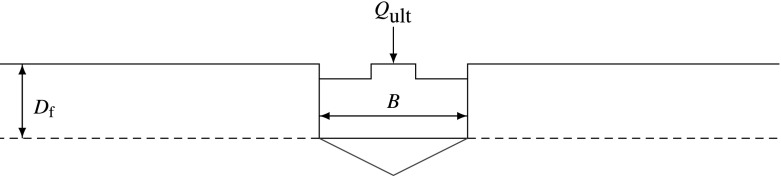
Punching

In dense dilating soil, the shear planes can reach the ground surface (Fig. 1). In loose soil, the shear planes, if any developed, would be confined to the surface of the rigid wedge (see Fig. 3). This mode of failure, which is more observed in loose clayey soil, is termed punching (Budhu 2000).

There is a plethora of equations available for estimation of bearing capacity of shallow foundations. Regarding the friction angle $\varphi$, cohesion *c* and surcharge *q*, one general expression for the ultimate bearing force $Q_{\mathrm {ult}}$ from the codes and standards can be summarized as follows (Wichtigsten Regelwerke 1991),

1 $$\begin{aligned} Q_{\mathrm {ult}}=B \cdot (\gamma \cdot B \cdot N_{\mathrm {b}} + c \cdot N_{\mathrm {c}} + q \cdot N_{\mathrm {d}}), \end{aligned}$$

where *B* is the breadth of the fundation, $\gamma$ the density of the soil and $N_{\mathrm {b}}, \ N_{\mathrm {c}}$ and $N_{\mathrm {d}}$ are dimensionless factors. $N_{\mathrm {b}}$ is the factor which takes into account the weight of the soil and is only a function of friction angle. $N_{\mathrm {c}}$ and $N_{\mathrm {d}}$ take into account, respectively, the effect of cohesion and surcharge. For a more detailed explanation of the factors, the reader is referred to Kolymbas (2017), Budhu (2000) and Leonards (1962).

## Experimental model test

The Soft PArticle Code (SPARC) is a newly developed method and like any other numerical method needs to be validated against experiments. For this purpose, the authors chose the experiments introduced in Aubram (2013). These experiments have been firstly designed and conducted for the validation of the arbitrary Lagrangian-Eulerian (ALE) method and are therefore appropriate for our purpose. Moreover, the experiments give an insight into the phenomenology of deformations caused by the settlement of shallow foundations.

### Test set-up

Aubram introduces in Aubram (2013) a set of test models, conducted in order to observe the displacement field during penetration into sand and also to investigate the load-settlement behavior of shallow foundations. The experiments have been carried out under quasi-static conditions and a wooden rectangular cuboid has been used as a model foundation, which almost guarantees plane strain deformation. The model provides a chamber with internal dimensions of $1003\hbox { mm }\times \,502\hbox { mm }\times 152\hbox { mm}$ filled with dry sand and the model foundation has dimensions of $150\hbox { mm }\times 100\hbox { mm }\times 150\hbox { mm }$, (breadth $B=150$ mm). The tests have been carried out at 1 g and no surcharge has been applied to the ground surface.

### Model test sand

The sand used for the experiments is a *fine-gravelly coarse sand* with diameter of 1–3 mm. The minimum and maximum void ratio of the sand lie between $e_{\mathrm {min}}=0.48$ and $e_{\mathrm {max}}=0.78$, respectively and the critical friction angle $\varphi _\mathrm {c}$ is equal to $31.5^{\circ }$. For more details concerning the model sand properties, the reader can refer to Aubram (2013). The material model applied for our simulation is the hypoplastic equation after von Wolffersdorff (1996).

### Details of penetration

The initially dense set-up of the experiment with $e_0=0.545$ is chosen for back-calculation in this study. For the dense set-up, a maximum relative penetration depth of $z_{\mathrm {max}}/B$ equal to 0.55 has been achieved in the experiment. The penetration rate for this experiment is $\varDelta z= 2 \ \mathrm {mm}$ per step.

## Numerical simulation and validation

### Geometry of the model

**Fig. 4:**
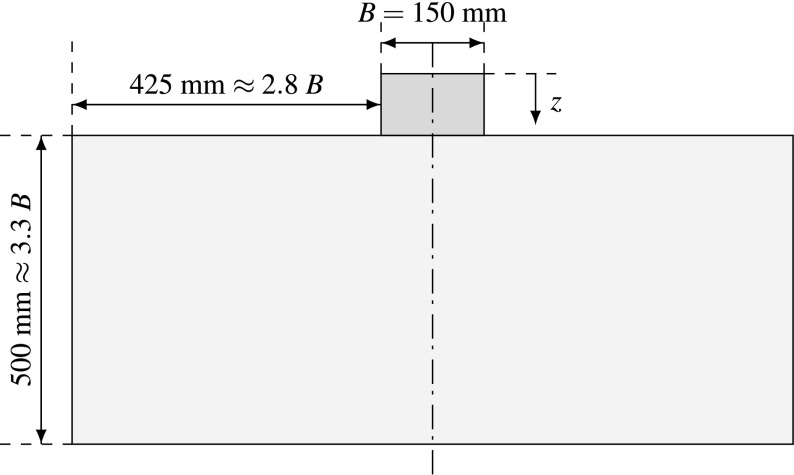
Initial geometry of the model Reproduced with permission from Bathaeian (2018)

The geometry of the numerical model is chosen in correspondence to the experiment with slight modifications (see Fig. 4). The simulation is conducted in plane strain conditions and although it is possible to take advantage of the symmetry, the complete model is simulated. In this way, in SPARC, the particles lying on the symmetry line, can benefit from a larger number of neighbors. This leads to better approximation of spatial derivatives and a more stable calculation procedure. However, for the demonstration of the results, the half symmetric part of the simulations is plotted.

### Foundation and boundaries

The flat-ended foundation which represents a strip foundation, is rigid and perfectly rough. The penetration is displacement controlled with a penetration increment of $\varDelta z = 0.3 \ \mathrm {mm}$ per time step. Particles lying on the walls of the model have freedom of movement in vertical direction and particles on the bottom of the model are free to move in horizontal direction.

On the ground surface, the static boundary condition,

2 $$\begin{aligned} \varvec{\sigma } \cdot \mathbf {n} -(-b) \mathbf {n}=\mathbf {0} \end{aligned}$$

is applied, where $\mathbf {n}$ is the normal vector of the surface and *b* is a constant pressure equal to 1 kPa for numerical stabilization, applied to the surface to avoid stress states in tension.

### Initial stress

The initial stress is assumed to be a $K_0$-state, with $K_0=0.47$. The stress grows linearly with depth,

3 $$\begin{aligned} \sigma _{zz}= & {} \gamma z, \end{aligned}$$

4 $$\begin{aligned} \sigma _{yy}= & {} \sigma _{xx}=K_0 \cdot \sigma _{zz}, \end{aligned}$$

with $$\gamma = 16.81 \ \mathrm {kN/m^3}$$ for $$e_0=0.545$$.

### Results and discussion

**Fig. 5:**
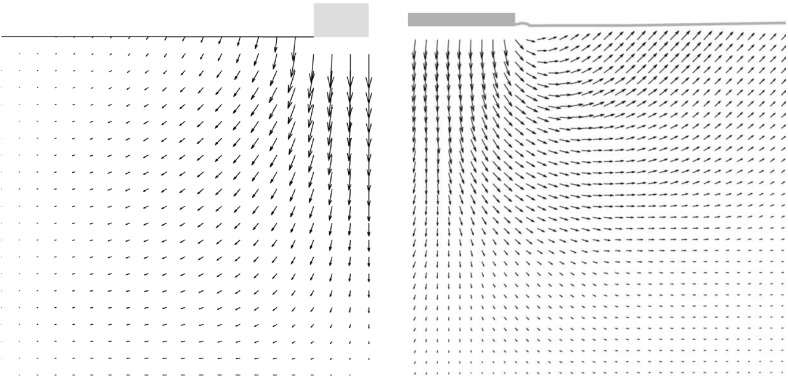
Qualitative comparison: left: SPARC simulation of shallow penetration into sand at $z/B=0.01$—smoothed incremental displacement field (Bathaeian 2018). Right: PIV result of shallow penetration into sand for the corresponding *z* / *B*-incremental displacements (see footnote 2) Figure adapted from Aubram (2013). (Verffentlichungen des Grundbauinstitutes der Technischen Universitt Berlin. Reproduced with permission of Shaker Verlag GmbH)

**Fig. 6:**
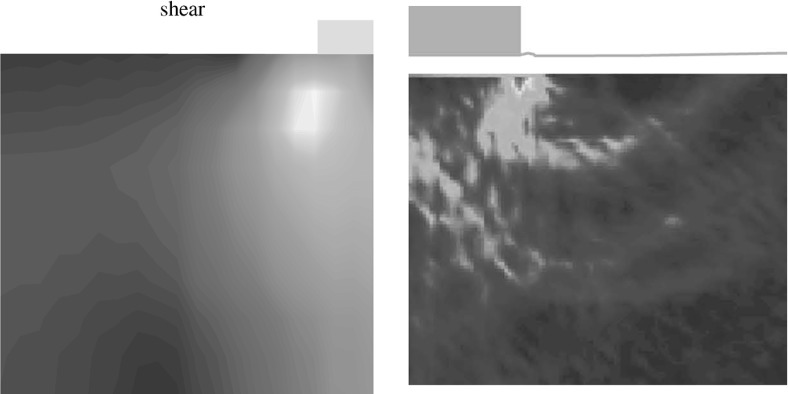
Qualitative comparison: left: SPARC simulation of shallow penetration into sand at $z/B=0.01$—smoothed maximum shear rate of deformation $\dot{\gamma }_{s}$ (Bathaeian 2018). Right: PIV result of shallow penetration into sand for the corresponding *z* / *B*—incremental maximum shear strain (see footnote 2) Figure adapted from Aubram (2013) (Verffentlichungen des Grundbauinstitutes der Technischen Universitt Berlin. Reproduced with permission of Shaker Verlag GmbH)

The incremental displacements after a small relative depth of $z/B=0.01$ are compared with experimental results in Fig. 5.^2^ SPARC is capable of simulating the outward movement of particles due to the penetrating foundation. However, the incremental displacements far away from the foundation and near to the surface of the ground are underestimated in comparison to those from the experiment. As discussed in Aubram (2013) by changing the values of the granular hardness $h_{s}$ [parameter of the hypoplastic model (von Wolffersdorff 1996)] a better prediction of the ground heaving is possible, however, this also influences the load-settlement behavior of the simulation and has therefore been avoided in this contribution. The maximum incremental shear strains of the experiments at $z/B=0.01$ are compared qualitatively in Fig. 6 with the maximum shear rates $\dot{\gamma }_{s}$ of deformation obtained from SPARC.

5 $$\begin{aligned} \dot{\gamma }_{s}=\frac{1}{2} |D_1 - D_2| \end{aligned}$$

where $D_1$ and $D_2$ are the maximum and minimum eigenvalues of the rate of the deformation tensor $\mathbf {D}$.^3^

**Fig. 7:**
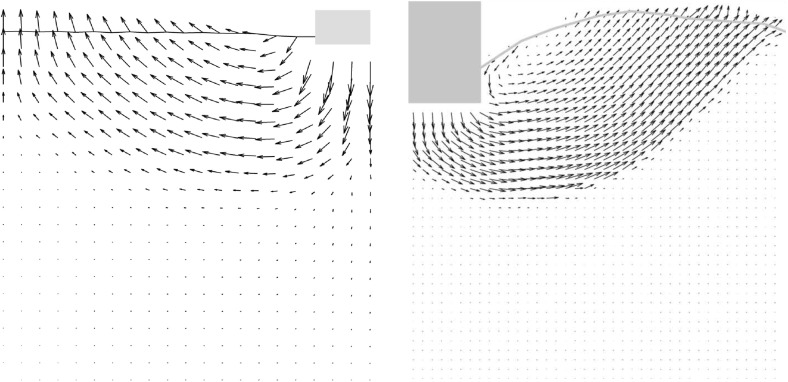
Qualitative comparison: left: SPARC simulation of shallow penetration into sand at $z/B\approx 0.18$—smoothed incremental displacement field (Bathaeian 2018). Right: PIV result of shallow penetration into sand for $z/B=0.33$—incremental displacements (see footnote 2) Figure adapted from Aubram (2013) (Verffentlichungen des Grundbauinstitutes der Technischen Universitt Berlin. Reproduced with permission of Shaker Verlag GmbH)

**Fig. 8:**
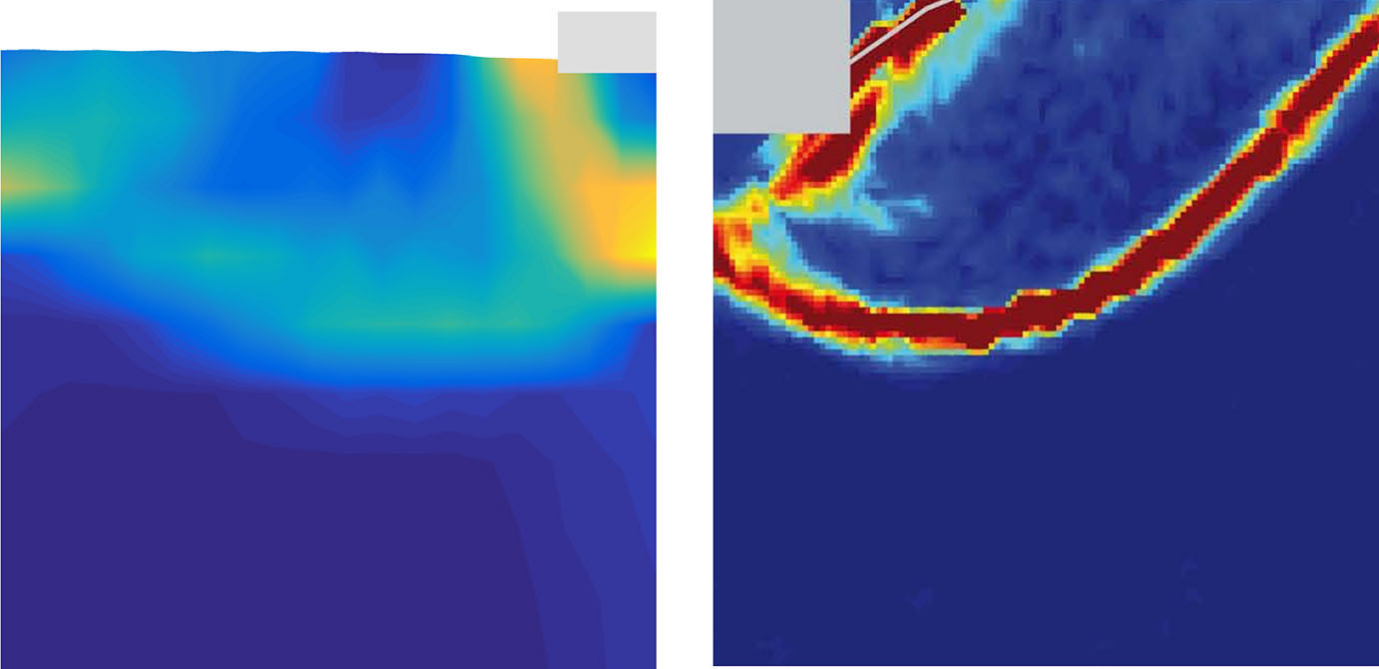
Qualitative comparison: left: SPARC simulation of shallow penetration into sand at $z/B\approx 0.18$—smoothed maximum shear rate of deformation $\dot{\gamma }_{s}$ (Bathaeian 2018). Right: PIV result of shallow penetration into sand for the $z/B=0.33$—incremental maximum shear strain (see footnote 2) Figure adapted from Aubram (2013) (Verffentlichungen des Grundbauinstitutes der Technischen Universitt Berlin. Reproduced with permission of Shaker Verlag GmbH)

**Fig. 9:**
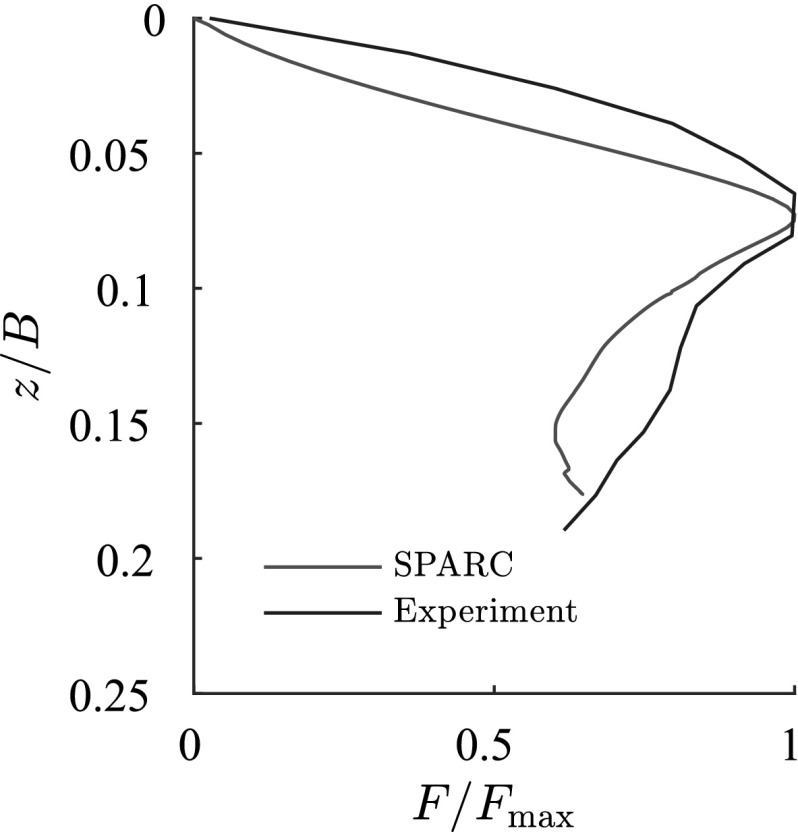
Comparison of load-settlement behavior for SPARC and the experiment (Bathaeian 2018), data of the experiment extracted from Aubram (2013)

**Fig. 10:**
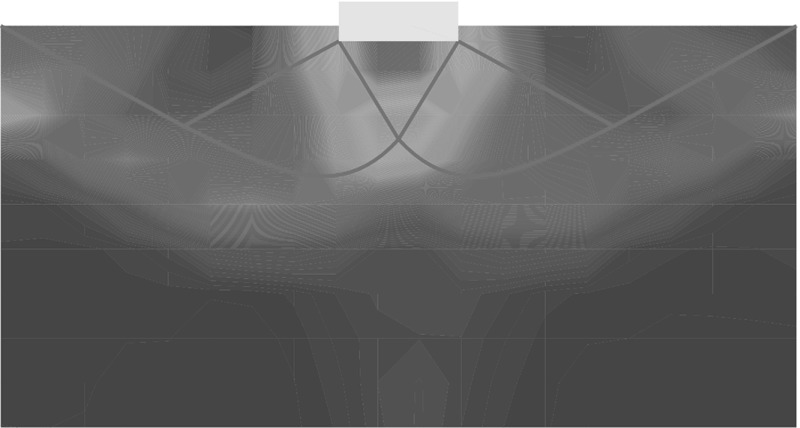
Comparison of the shape of shear zones according to the analytic solution demonstrated in Fig. 1 with the prediction of SPARC Reproduced with permission from (Bathaeian 2018)

PIV results show that initially the shear strains localize beneath the outer edge of the foundation, starting to form a wedge under the foundation as discussed in Sect. 2. Results of SPARC also show the formation of the wedge beneath the foundation initiated from the outer edge of the foundation. The experimental results show that radial shear bands start to develop from the very beginning of the penetration. However, these initial radial shear bands are not reproduced by SPARC in this stage of penetration. In Fig. 7, the incremental displacements after reaching the peak of the load-settlement curve and complete formation of shear zones are plotted. Particles beneath the foundation are pushed aside and to the surface due to the approaching foundation, which is very well simulated by SPARC. The incremental displacements of SPARC near to the wall boundary show that the particles move almost vertically upwards and the horizontal components of the displacement disappear, which does not correspond to the results from the experiment. Results from Fig. 8 show how well SPARC is capable of reproducing the failure mechanism and the development of the three main components of failure, namely the wedge and the shear zones, c.f. Fig. 2. The dense sand with an initial void ratio $e_0=0.545$, loosens in the shear bands and reaches a maximum value of $e\approx 0.61$. In the wedge beneath the foundation, the material is on the other hand densified and reaches $e \approx 0.52$. Evaluation of incremental volumetric strain of the experiments in Aubram (2013), p. 291, show that no extreme densification occurs in the wedge below the foundation, which also corresponds to the prediction of SPARC in comparison to the predictions made by the ALE method in Aubram (2015) where a densification up to $e=0.482$ in the wedge has been predicted. This is due to the fact that SPARC can more realistically simulate the outward escape of the particles due to penetration under the foundation than FE and therefore no excessive densification is predicted. The normalized load-settlement curve obtained by SPARC is compared with the experiment in Fig. 9. The results show that SPARC has been able to predict the load-settlement behavior well and the peak of the curve is predicted at the same relative penetration *z* / *B* of the experiment. This can be attributed to the more realistic simulation of particle trajectories in the wedge area and their outward movement which result in an earlier prediction of the full mobilization of the shear strength in comparison to other mesh based methods [e.g. see Aubram (2015)]. Secondly, no excessive densification is predicted by SPARC in the wedge which also contributes to a better prediction of the load-settlement behavior. In Fig. 10, the shear bands obtained by the analytical solution as explained in Fig. 1 are plotted over the shear bands obtained by SPARC for comparison. It can be seen that the depth of the wedge obtained from the analytical solution is in good agreement with the one obtained from SPARC, however, the shear bands predicted by SPARC lie deeper in comparison.

**Fig. 11:**
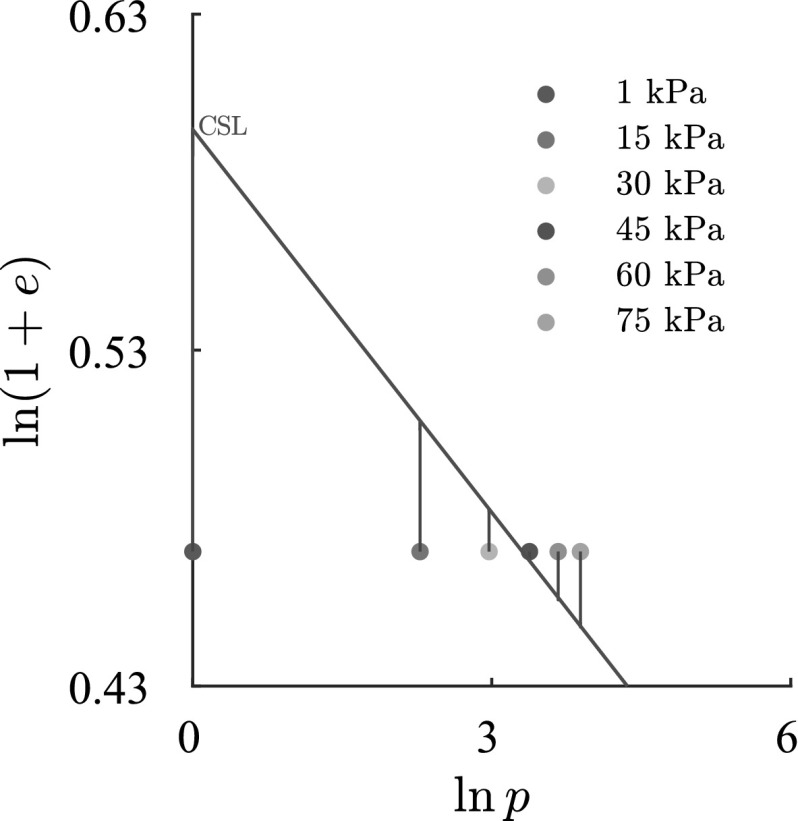
Demonstration of initial conditions ($p - e$) for each simulation (the colorful points) and void ratio evolution assuming a constant mean pressure *p*, where $p=\frac{1}{3}(\sigma _{1} + \sigma _{2} + \sigma _{3})$

## Shallow penetration into clay

For the simulation of shallow penetration into clay the material model barodesy has been used. Barodesy for clay (Medicus and Fellin 2017), in contrary to the popular elastoplastic material model, Mohr - Coulomb, takes into account effects, resulting from changes in void ratio *e* and mean pressure *p* and is therefore more suitable for modeling geotechnical problems such as shallow penetration, by which excessive changes in void ratio and stress state occur due to the penetration, for more details see Bode (2017). In order to conduct a thorough investigation, the simulations are carried out for six increasing surcharges from 1 to 75 kPa, which equal overburdens from $D_f \approx 0 \sim 4.5$ m, respectively. Meanwhile, the initial void ratio for all six simulations is constant and equal to $e_0=0.6$. In Fig. 11, the relationship for the corresponding surcharges and their relation to the ciritical state line (CSL) are plotted. It is apparent that the lower three surcharges lead to an over-consolidated initial behavior, whereas the upper two surcharges lead to a normal-consolidated initial behavior. The surcharge of 45 kPa initiates quasi from the critical state. For the surcharges 1–30 kPa, the material behaves dilatant to reach the critical state line. On the contrary, for the surcharges 45–75 kPa, a contractant behavior due to shearing is expected.

**Fig. 12:**
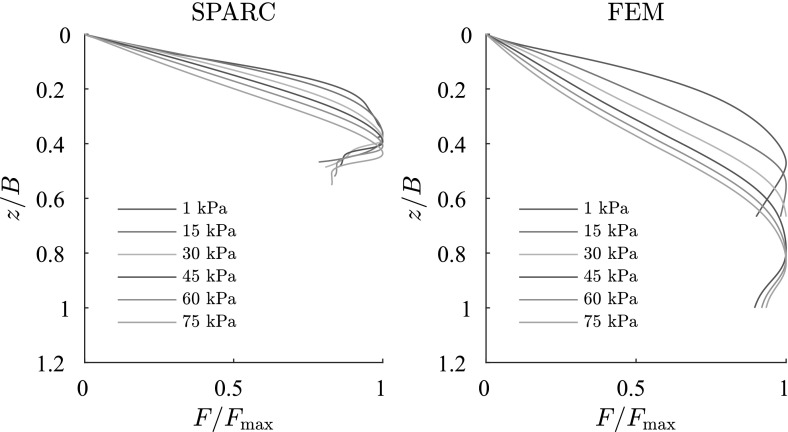
Comparison of normalized load-settlement behavior for SPARC and FEM for different surcharges

In Fig. 12, normalized results of load-settlement behavior of SPARC and FEM are compared. Results show that for larger surcharges, the peak of the load-settlement curve is achieved at a deeper depth. This behavior is expected, since for larger surcharges, the developed shear zones must push heavier loads upward and consequently larger deformations are demanded. One main difference between the results of FEM and SPARC to notice, is that FEM predicts in general the peak at a deeper relative penetration in comparison to SPARC.

### Low surcharge, $q=1$ kPa

**Fig. 13:**
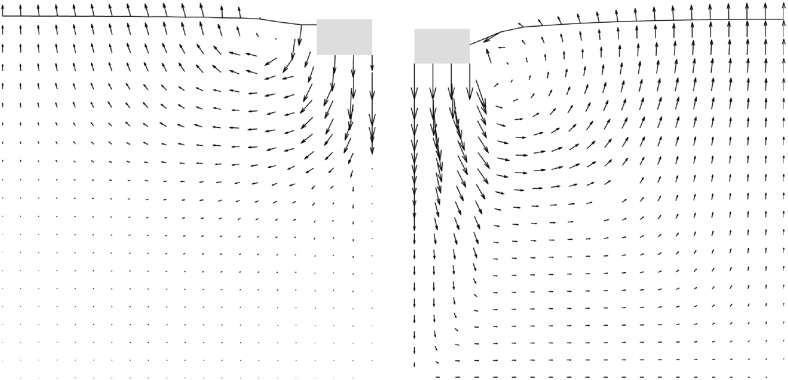
Qualitative comparison, left: SPARC simulation of shallow penetration into clay for $q=1$ kPa—smoothed incremental displacement field. Right: FEM simulation of shallow penetration into clay—smoothed incremental displacement field

In Fig. 13, the incremental displacements after the peak of the load-settlement curve are plotted. The displacements under the foundation for SPARC disappear in a higher depth compared with FEM. The displacements near to the surface for both methods look similar, however, the settlements near to the foundation for FEM are larger. A closer look at the lower edge of the foundations shows, that the upward displacements for SPARC are larger in this area in comparison to FEM which could explain the predicted excessive settlements near to the foundation by FEM. Furthermore, the incremental displacement field for the FEM is influenced by the geometry of the model. This effect is not apparent for results of SPARC.

**Fig. 14:**
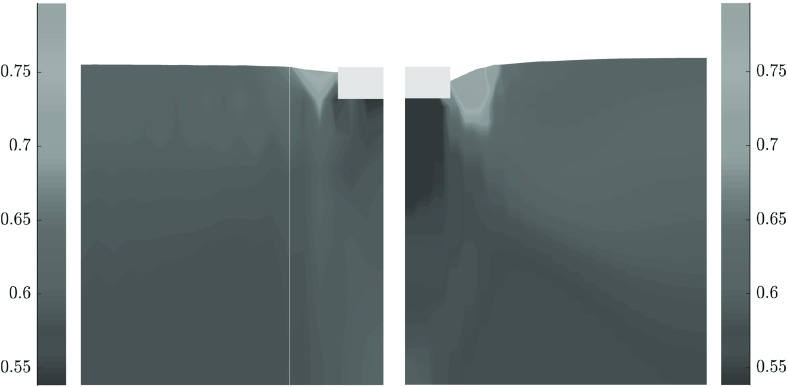
Left: SPARC simulation of shallow penetration into clay for $q=1$ kPa—void ratio field. Right: FEM simulation of shallow penetration into clay—void ratio field

In Fig. 14, the field of void ratio after the peak for both methods is compared. In the first glance, one notices the excessive densification under the foundation modeled with FEM. The deformation predicted by FEM under the foundation resembles somewhat the oedometric condition. However, the void ratio under the foundation for SPARC has not excessively decreased and mainly under the middle of the foundation a slight densification can be detected.

Shear bands for simulations with clay follow a different pattern as those for sand and pose a punching shape at the corners of the foundation, this is because the settlements and deformations for clay are in comparison to sand larger . This means that for clay, the shearing occurs mainly on the sides of the foundation and relatively close to the surface, in comparison to sand, where the shear zones develop mainly in the depth and reach the surface.

### High surcharge, $q=75$ kPa

**Fig. 15:**
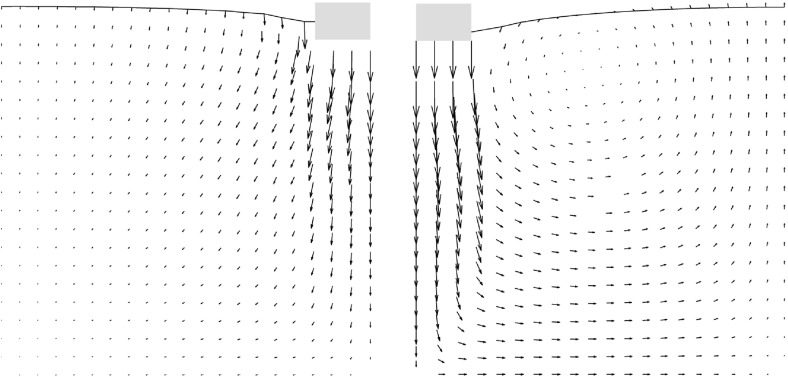
Qualitative comparison, left: SPARC simulation of shallow penetration into clay for $q=75$ kPa—smoothed incremental displacement field. Right: FEM simulation of shallow penetration into clay—incremental displacement field

**Fig. 16:**
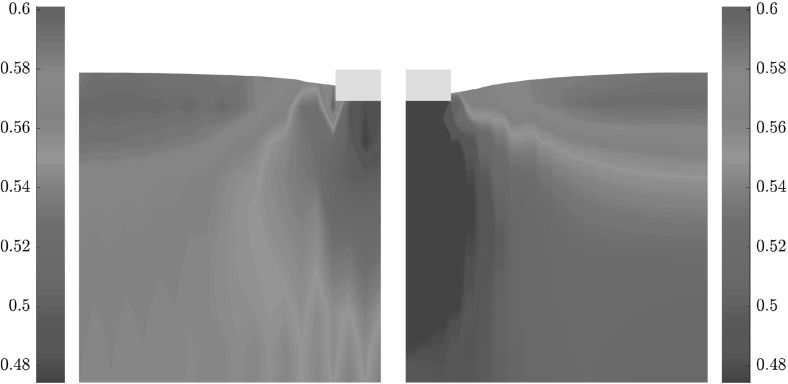
Left: SPARC simulation of shallow penetration into clay for $q=75$ kPa—smoothed void ratio field. Right: FEM simulation of shallow penetration into clay—void ratio field

In Fig. 15 the incremental displacements after the peak are compared. The vertical incremental displacements under the foundation for FEM do not vanish and continue to the bottom of the model, which leads to an excessively densified column under the foundation (see Fig. 16). In comparison to Fig. 13, the incremental displacements on the surface for both methods are smaller than those for the lower surcharge ($q=1$ kPa). This could be attributed to I) higher surcharge and II) the contractant behavior of the material as demonstrated in Fig. 11. The distribution of void ratio is plotted in Fig. 16. As discussed earlier, denisfication below the foundation in SPARC is smaller in comparison to FEM. As opposed to FEM, the punching shear zone near to the corner of the foundation predicted by SPARC, can still be detected for the higher surcharge.

## Discussion of results for clay

**Fig. 17:**
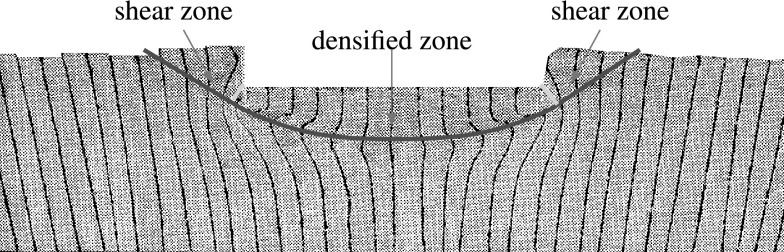
Deformation of loose clay caused by penetration of a circular foundation Figure adapted from Tani and Craig (1995)

There are not many experimental tests available which investigate the behavior of clay under the deformations induced by shallow foundations. In Fig. 17, we present the deformations caused by the penetration of circular foundation into clay (Tani and Craig 1995). Figure 17 shows that in contrast to sand, the deformations are mainly concentrated under the foundation in form of densification and near to the walls of the foundation in form of shear zones, which are in good agreement with the pattern of deformations and shear zones in Figs. 14 and 16.

In Table 1, densification under the foundation for the same relative depth of each method is compared. As expected, the results show that the values for FEM are about 35–45% larger in comparison to SPARC. It is also interesting to notice that in case of lower surcharge $q=1$ kPa (overconsolidated material) the changes in the void ratios are considerably smaller than the changes in case of higher surcharge ($q=75$ kPa), where the soil is considered as normal- consolidated.

**Table 1:** Comparison of average densification (changes in void ratio) under the foundation for the same relative depths

	Surcharge $q=1$ kPa (%)	Surcharge $q=75$ kPa (%)
SPARC	9	17
FEM	13	23

**Fig. 18:**
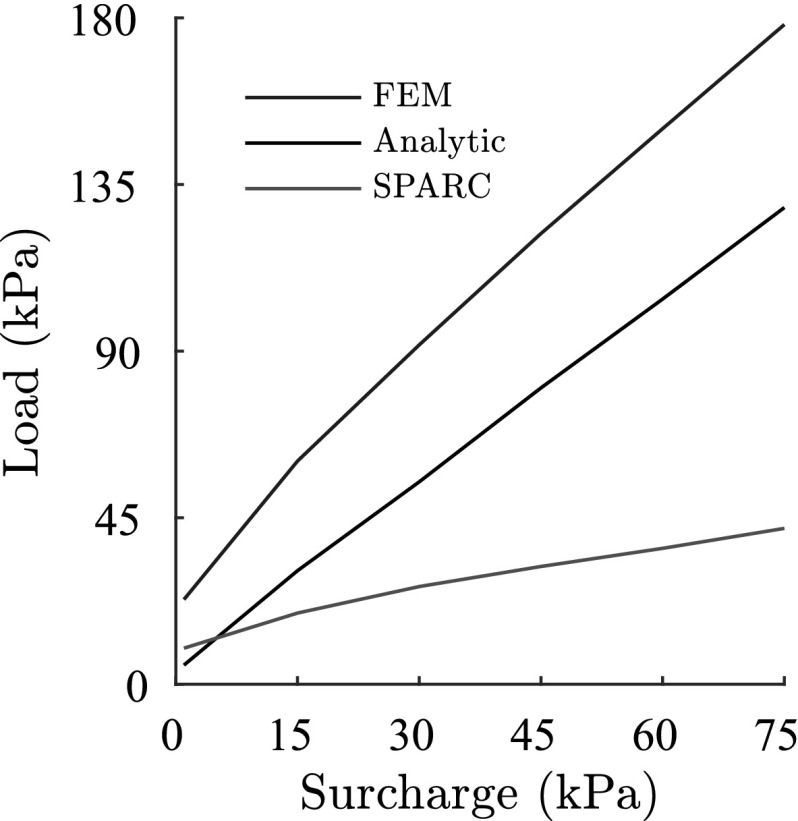
Comparison of bearing capacity for SPARC and FEM with analytical solution after Bowels in Bowles (1974)

In Fig. 18 the maximum bearing capacities predicted by SPARC and FEM for each surcharge are compared with the analytical solution proposed in Bowles (1974) for strip foundations. For the calculation of the analytical values corresponding to each surcharge, the friction angle $\varphi$ and cohesion *c*, are determined in proportion to the the stress state and void ratio under the foundation at the peak of the load-settlement curve [$\varphi$ and *c* are determined by the same procedure explained in Schneider-Muntau et al. (2017)].

SPARC predicts lower bearing capacities than the analytical solution for the corresponding surcharges and FEM overestimates the bearing capacities significantly. As discussed earlier, the overestimation of FEM can be attributed to the excessive densification of the material beneath the foundation. However, the reason for the underestimation of the results of SPARC in comparison to the analytical solution could be that the shape of the shear zones for clay do not completely comply to the assumed shear zones introduced in Fig. 1. Therefore, the bearing capacity obtained for the assumed shear zones in Fig. 1 have higher values, since the area of the shear zones are larger in comparison to those acquired for clay. However the “real” values of bearing capacities in nature are unknown.

## Conclusion

Simulation of large deformations due to penetration is still a challenging task. Mesh based methods have to deal with large mesh deformations and distorted meshes. Mesh free methods are known to perform better when it comes to simulation of problems associated with large deformations. Simulation of deformations caused by settlement of shallow foundations is considered as one of the geotechnical problem associated with large deformations. In this study the results of small scale experiment of shallow foundations on sand by Aubram are compared with results of the mesh free approach SPARC for validation. SPARC turned out to be able to simulate the deformations caused by settlement of shallow foundations. The comparisons with small scale experiment on sand by Aubram have shown a satisfactory prediction of the relative depth of settlement at peak. Moreover, mesh based methods yield a higher value for the bearing capacity (peak of the load-settlement behavior). Results of densification of the material beneath the foundation predicted by SPARC are more comparable to the experimental evaluations. As opposed to SPARC, the FEM results predict higher densification for the elements beneath the foundation which are not confirmed by the experiments.

The predicted failure mechanism is dependent on the density (void raio) and average mean pressure in soil. For dense sand, the shear bands obtained by SPARC are qualitatively comparable to the analytic solution and are also in good agreement with the experiments. In this context, it is important to highlight, that the analytical approaches only give a very simplified solution for bearing capacity and failure mechanism of shallow foundations as the deformations and compaction of the underlying soil are not taken into account.

Additionally, a more detailed comparison of mesh based and mesh free methods has been performed on a similar geometry for clay, using the material model, “barodesy for clay”. The settlements in clay at peak are obviously larger than the settlements in sand due to the softer behavior of clay, also the failure pattern is different. For clay more a punching failure mechansim is observed which is in good agreement with field observations.

SPARC simulations also show that smaller boundaries are required for the numerical model, since the incremental displacements disappear before reaching the bottom and side walls. However, the incremental displacements predicted by FEM do not disappear at the limits of the model of the same dimensions used for SPARC.

SPARC has turned out to be the method of choice for simulating deformations caused by settlement of shallow foundations. The validation with experimental results on sand and the comparison with numerical simulations and analytical approaches on clay show good accordance and confirm the good performance of SPARC for simulation of geotechnical problems, where the conventional mesh based methods seem to reach their limits.

As only very few case studies on the settlement of shallow foundations have been conducted, especially for clayey soil. It is recommended to consider conducting experimental tests of settlement of shallow foundations on clay. These tests can be beneficial for validation of both numerical methods and also material models.

## Footnotes

1 Particle image velocimetry.

2 Differences in the size of the foundations in Figs. 5, 6, 7 and 8 are due to the scaling factors applied to the figures in the experimental results in Aubram (2013).

3 Rate of deformation tensor $\mathbf {D}$, is the symmetric part of the velocity gradient.
